# Polymorphism in the Hypoxia-Inducible Factor 1alpha Gene May Confer Susceptibility to LDD in Chinese Cohort

**DOI:** 10.1371/journal.pone.0073158

**Published:** 2013-08-26

**Authors:** Wen-Ping Lin, Xue-Jin Wang, Cong-Ren Wang, Li-Qun Zhang, Neng Li, Fa-Sheng Wang, Jian-Hua Lin

**Affiliations:** 1 Department of Orthopedic Surgery, The Second Affiliated Hospital, Fujian Medical University, Quanzhou, Fujian Province, People’s Republic of China; 2 Department of Obstetrics and Gynecology, The Second Affiliated Hospital, Fujian Medical University, Quanzhou, Fujian Province, People’s Republic of China; 3 Department of Oncosurgery, Affiliated Quanzhou First Hospital, Fujian Medical University, Quanzhou, Fujian Province, People’s Republic of China; 4 Department of Orthopedic Surgery, The First Affiliated Hospital, Fujian Medical University, Fuzhou, Fujian Province, People’s Republic of China; 5 Department of Pathogeny Biology, College of Preclinical Medicine, Fujian Medical University, Fuzhou, Fujian Province, People’s Republic of China; Centers for Disease Control and Prevention, United States of America

## Abstract

**Objective:**

This study aimed to investigate whether or not hypoxia-inducible factor-1α (HIF-1α) gene variants are associated with the susceptibility and clinical characteristics of lumbar disc degeneration (LDD).

**Methods:**

We examined 320 patients with LDD and 447 gender- and age-matched control subjects. We also determined the HIF-1α gene variants, including C1772T (P582S) and G1790A (A588T) polymorphisms.

**Results:**

Significant differences were observed in allelic and genotypic distributions of 1790 A > G polymorphisms between LDD cases and control subjects. Logistic regression revealed that 1790 AA genotypes indicated a protective effect against the development of LDD. The HIF-1α 1790 A > G polymorphisms also affected the severity of LDD as evaluated based on the modified Japanese Orthopedic Association (mJOA) scores. The 1790 AA genotype carriers exhibited significantly lower mJOA scores than AG and GG carriers. C1772T did not show any association with the risk and severity of LDD.

**Conclusion:**

Our study suggested that HIF-1α 1790 A > G polymorphisms may be used as a molecular marker to determine the susceptibility and severity of LDD.

## Introduction

Lumbar disc degeneration (LDD) is a major cause of disability and has been associated with low back pain [1–4]. LDD is caused by various intrinsic and extrinsic factors; however, the etiology of LDD remains poorly understood. Environmental factors such as age, gender, smoking habits, overweight, and exposure to vibration may contribute to LDD [3,5–7]. Studies have also revealed that the genetic background may contribute to the development of LDD [8,9].

The lumbar disc tissue is avascular, although small discrete capillary beds are found in the dorsal and ventral surfaces. For this reason, nucleus pulposus cells commonly reside in a hypoxic environment [10]. The functions of hypoxia-inducible factor-1α (HIF-1α) and other factors associated with hypoxia in the mechanism of LDD development has also been reported [11].

HIF-1α functions as a key regulator of cellular responses to hypoxia and other processes, including angiogenesis, erythropoiesis, energy metabolism, vasomotor function, and apoptotic/proliferative responses [12–14]. HIF-1α is expressed in nucleus pulposus cells and has an important function in regulating energy metabolism and matrix synthesis [15–17]. Another study has revealed that HIF-1α may function in the survival of disc cells and the resorption of the herniated disc in human intervertebral discs [18].

HIF-1α expression is regulated by gene polymorphism. In the HIF-1α gene, two polymorphisms, namely, C1772T (P582S) and G1790A (A588T) polymorphisms significantly increase gene transcriptional activity; these polymorphisms are correlated with the increased expression of proteins compared with the wild-type sequence HIF-1α C1772T polymorphism, thereby leading to HIF-1α mRNA overexpression in patients with prostate cancer [19,20].

Although HIF-1α expression has been observed in nucleus pulposus cells, the function of HIF-1α gene polymorphisms in LDD remains unknown. We postulated that the HIF-1α gene variants may be related to the risk of LDD. In the present study, the patients with LDD and the healthy control subjects were enrolled to investigate whether or not HIF-1α gene variants are associated with the susceptibility and clinical characteristics of LDD.

## Materials and Methods

### Ethics statement

This study was approved by the Ethical Committee of Fujian Medical University (No. 2012-2-56). All of the participants provided their written informed consent to participate in this study.

### Subject Enrollment

We examined 320 patients with LDD and 447 gender- and age-matched control subjects from September 2008 to August 2012 in the affiliated hospital of our school. All of the patients received MRI evaluation, and all of the control subjects were evaluated by CT or MRI. The diagnosis of LDD was confirmed by two orthopedic surgeons specializing in spinal diseases, but these surgeons were unaware of the study design. The disc degeneration grade was determined according to Schneiderman’s classification of MRI [21]. The exclusion criteria were listed as follows: a) subject with autoimmune and inflammatory diseases; b) severe degenerative disc disease with a collapsed intervertebral space; c) previous surgical treatment in the lumbar spine; d) severe osteoporosis; and e) segmental instability. The severity of LDD was evaluated based on the modified Japanese Orthopedic Association (mJOA) scoring system [22]. The following data were collected from the participants: gender; age; body mass index (BMI); smoking status; and family history of LDD.

### HIF-1α genotyping

Genomic DNA was isolated from the peripheral blood leukocytes according to standard protocols. Polymerase chain reaction (PCR) was performed to amplify the 178 bp fragment of exon 12 in the HIF-1α human gene by using 5ʹ-CAT GTA TTT GCT GTT TTA AAG-3ʹ as the forward primer and 5ʹ-GAG TCT GCT GGA ATA CTG TAA CTG-3ʹ as the reverse primer. The mixture (30 µL) used for PCR contained 200 ng of the template DNA, 0.2 mM of each dNTP, 0.5 µM of each forward and reverse primer, 1.5 mM of MgCl_2_, 0.5 U of Taq polymerase, and 3 µL of 10× PCR buffer. The PCR conditions were listed as follows: denaturation at 95 °C for 5 min; 35 cycles of denaturation at 95 °C for 30 s; annealing at 61 °C for 30 s; extension at 70 °C for 1 min; and a final extension at 72 °C for 10 min. The PCR products were purified and sequenced using the Big Dye Terminator kit (version 3.1) on an ABI Prism® 3100 Automated DNA sequencer according to the manufacturer’s protocol (Applied Biosystems, Foster City, CA).

### HIF-1α and VEGF Western blot

A total of 134 herniated lumbar intervertebral discs were collected during surgery from the patients with LDD and who underwent surgical treatment. The samples (0.3 g to 0.5 g) were homogenized and lysed. The extracts were resolved on sodium dodecyl sulfate (SDS)-polyacrylamide gels and then transferred to nitrocellulose membranes (BioRad). The proteins were also resolved by electrophoresis on 8% to 12% SDS-polyacrylamide gels and then transferred to polyvinylidene difluoride membranes (Bio-Rad) by electroblot. The membranes were blocked with 5% non-fat dry milk in TBST (50 mM Tris, pH 7.6, 150 mM NaCl, and 0.1% Tween 20) and incubated overnight at 4 °C in 3% non-fat dry milk in TBST with anti-Hsp70 (Stressgen, 1:4000), anti-HIF-1α (Novus Biological, 1;1000), VEGF (Santa Cruz,1:1000), and anti-GAPDH (Santa Cruz, 1:2000) antibodies. Immunolabeling was detected using an ECL reagent (Amersham Biosciences).

### Statistical analysis

For each SNP, a chi-square test was performed to assess Hardy-Weinberg equilibrium and the association between the LDD cases and the control groups based on allelic and genotypic frequencies. The characteristics of the LDD subjects and the control subjects were compared by performing chi-square or Student’s *t*-test according to the variable types. Binary logistic regression test was performed to determine the risk factor of LDD. The odds ratios (OR) and 95% confidence intervals (CIs) were calculated by adjusting age, gender, smoking status, and other clinical data. Results were considered significantly different at P < 0.05. The Raytest TINA software (http://www.raytest.de/service/raytest_catalog.html) was used for western blot to perform densitometric analysis and evaluate the expressions of HIF-1α and VEGF as described previously [23]. Differences in the relative expressions among different genotypes were compared by ANOVA in SPSS software (Statistical Package for the Social Sciences, version 16.0, SPSS Inc., Chicago, IL, USA).

## Results

### Characteristics of the cases and the control subjects

Table 1 shows the demographic characteristics of the LDD subjects and the control subjects. Gender, age, and BMI were similar between the two groups. Smokers were more common in the LDD subjects than in the control subjects (39.2% vs. 23.3%, P = 0.041). The patients with LDD exhibited a higher percentage of DM than the control subjects (P = 0.033). The incidence of positive family history was markedly higher in the LDD group than in the control group (18.3% vs. 8.3%, P < 0.001).

**Table 1:** Characteristics of subjects.

**Variables**	**Case n=320**	**Control n=447**	**P**
Age(mean ± SD)	47.1 ± 7.2	47.0± 6.8	0.584
Gender (Male, %)	154 (48.1%)	213 (47.6%)	0.743
BMI(mean ± SD)	23.7 ± 3.5	23.5 ± 2.7	0.772
Smoking (%)	125 (39.1%)	104 (23.3%)	0.041
Family history (%)	58 (18.4%)	47 (8.3%)	< 0.001
History of labor work (%)	120 (37.5%)	51 (11.4%)	< 0.001
DM	62 (19.4%)	45 (10.1%)	0.0033

Table 2 summarizes the allelic frequencies and genotypic distributions of the SNPs in the LDD cases and the control subjects. The genotypic distributions of each SNP were consistent with Hardy-Weinberg equilibrium (P > 0.05). For SNPs at 1772 C > T, no statistically significant difference was observed in the distribution of the alleles and genotypes among the patients and the control subjects (P = 0.982 and P = 0.482, respectively). For SNPs at 1790 G > A, the AA prevalence was markedly lower in the LDD cases than in the control subjects (20.80% vs. 52.49%, P = 0.001). Allele analysis revealed that the A allele frequencies were 43.25% and 48.5% in the patients with LDD and the control subjects, respectively (P = 0.001). Using GG as the reference, we conducted multivariate logistic regression analysis and found that the AA genotype carriers exhibited lower risks of developing LDD (adjusted OR = 2.15, 95% CI = 1.30–3.11, adjusted P = 0.002), in which age, gender, BMI, smoking status, family history, history of labor, and DM were adjusted.

**Table 2:** The genotype and allele distribution of HIF-1α gene SNPs in LDD cases and controls.

	LDD		Control						
	N	%	N	%	global P	adjusted OR	95%CI		adjusted P
1772CC	90	32.85%	101	33.55%	0.982	1			
1772CT	143	52.19%	156	51.83%		1.03	0.72	1.48	0.88
1772TT	41	14.96%	44	14.62%		1.05	0.63	1.74	0.86
C	323	58.94%	358	59.47%	0.452	1			
T	225	41.06%	244	40.53%		1.02	0.81	1.29	0.86
1790GG	94	34.31%	76	25.25%	0.011	1			
1790GA	123	44.89%	134	44.52%		0.74	0.5	1.1	0.13
1790AA	57	20.80%	91	30.23%		0.51	0.32	0.79	P<0.001
G	311	56.75%	286	47.51%	0.001	1			
A	237	43.25%	316	52.49%		0.69	0.55	0.87	P<0.001

**Figure 1:**
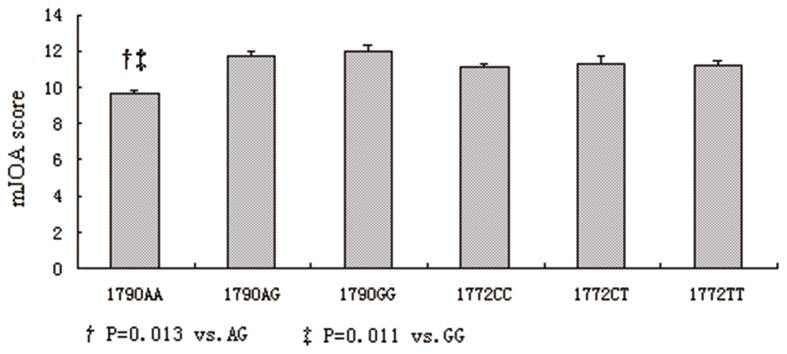
HIF-1α SNPs and the LDD severity within LDD subjects.

We analyzed the association of HIF-1α SNPs and the severity of LDD in the LDD subjects. The severity of LDD was stratified according to HIF-1α genotypes. Figure 1 shows that the severity of LDD was similar in the subjects with 1772 C > T genotype (P > 0.05). However, the severity of LDD in the subjects with 1790 A > G genotype were significantly different. The genotype carriers of 1790 AA exhibited significantly lower mJOA scores (9.6±0.2) than AG and GG carriers (11.7±0.2 and 12.0±0.3, respectively).

**Figure 2:**
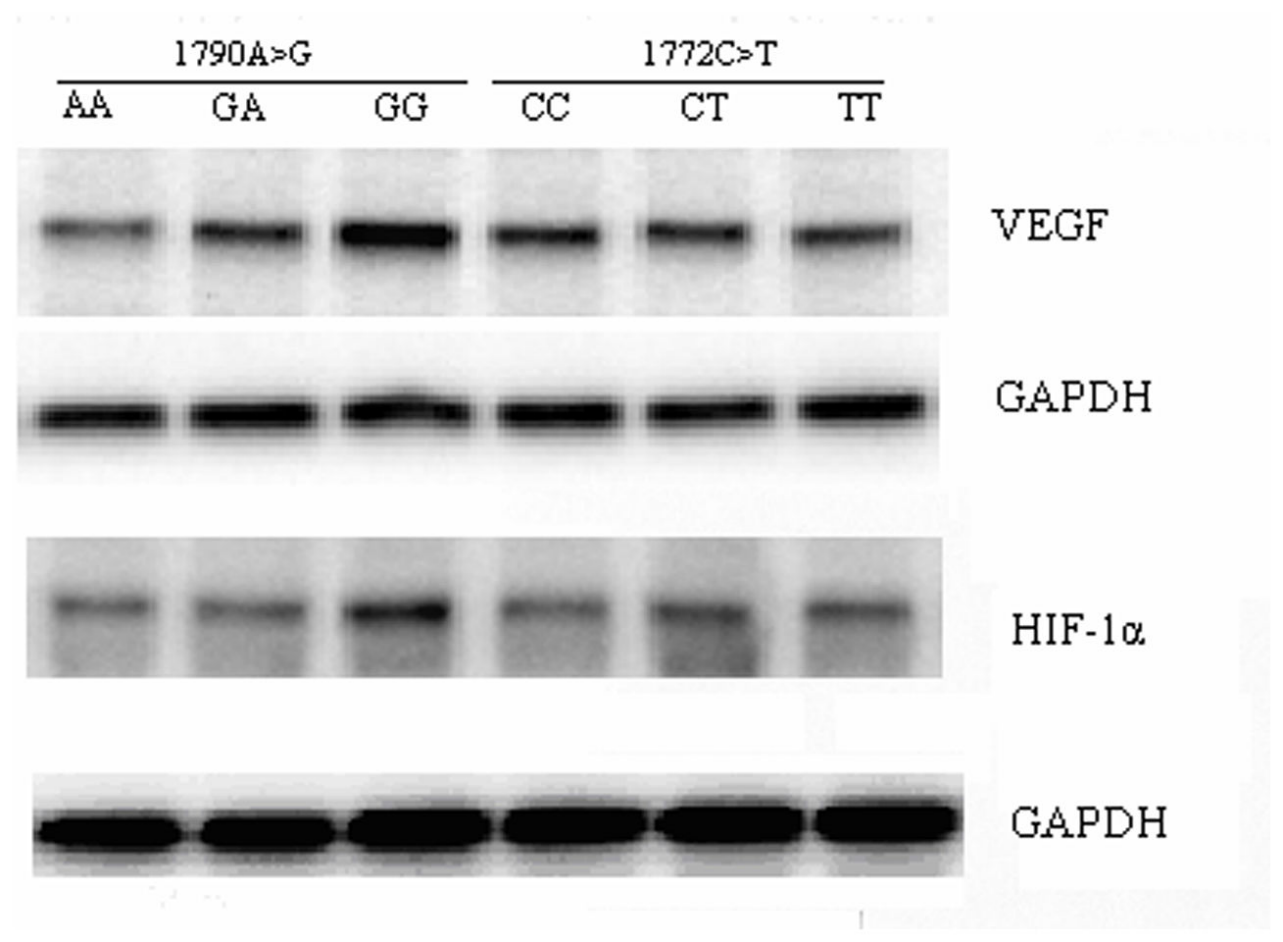
HIF-1α and VEGF protein expression in samples from LDD patients.

**Figure 3:**
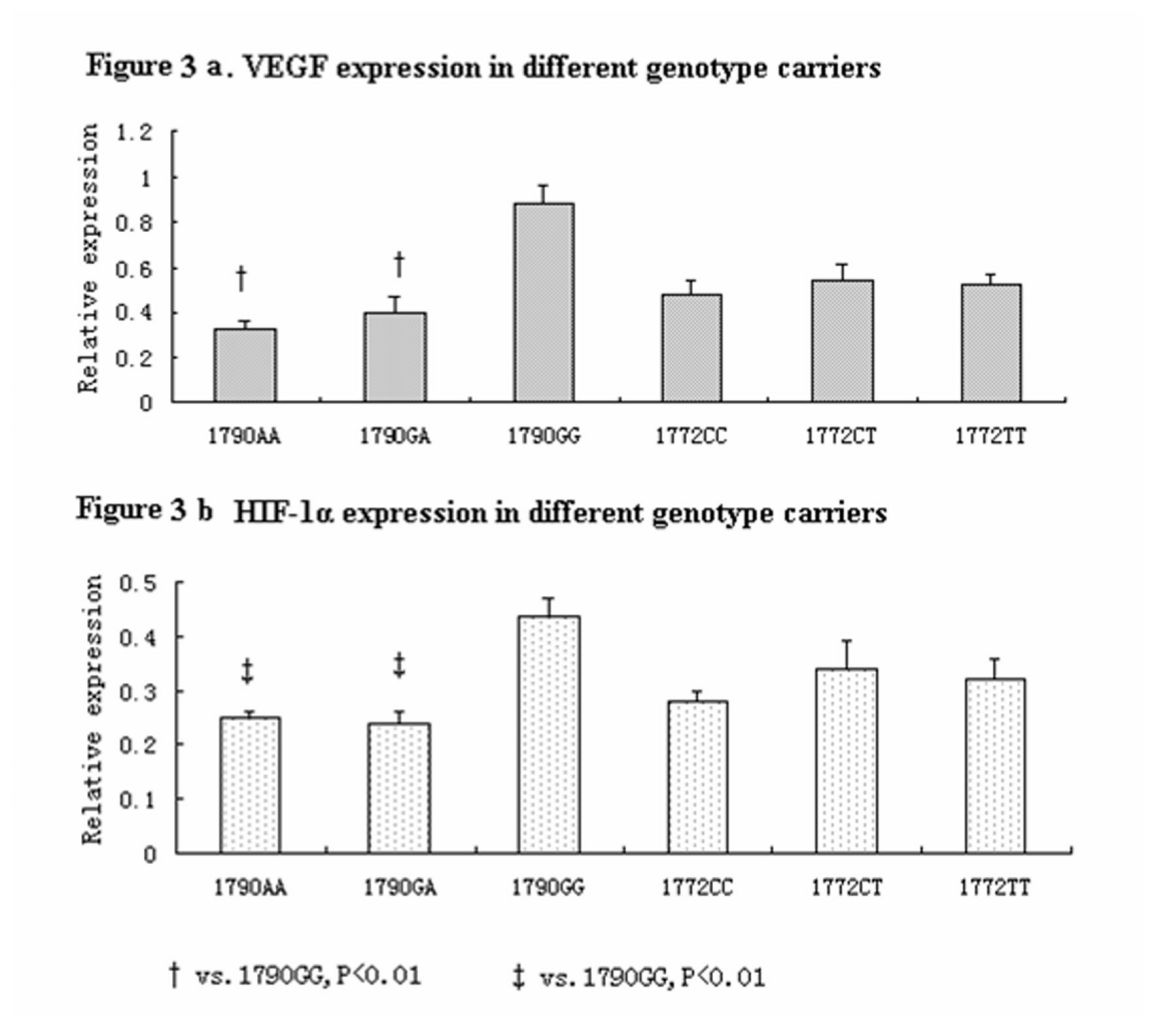
The VEGF and HIF-1α relative expressions by 1790G>A genotypes.

We compared HIF-1α and VEGF protein expressions in the samples obtained from the patients with LDD. The Characteristics and genotype distribution for 134 subjects selected for western blot were shown in Table S1 and Table S2. The herniated lumbar intervertebral discs collected from the patients with LDD exhibiting different genotypes underwent surgical treatment. Figure 2 shows the typical bands of the samples from the patients with LDD. We detected the HIF-1α protein expression by western blot analyses. Our results showed that the 1790 A > G SNPs significantly affected HIF-1α and VEGF expressions. The HIF-1α and VEGF protein expressions were significantly lower in 1790 AA carriers than in 1790 AG and 1790 GG carriers. VEGF expression was lower in 1790 AA carriers than in 1790 AG and 1790 GG carriers. By contrast, 1772 C > T genotype did not exhibit any change in HIF-1α and VEGF protein expressions. Quantitative analyses by Raytest TINA software showed that the relative protein expressions of VEGF were 0.33±0.03, 0.40±0.07, and 0.88±0.06 in 1790 AA, 1790 GA, and 1790 GG carriers, respectively (P < 0.001, Figure 3a). The relative expressions of HIF-1α were 0.25±0.01, 0.26±0.02, and 0.43±0.03 in 1790 AA, 1790 GA, and 1790 GG carriers, respectively (P < 0.001, Figure 3b). These results indicated that the relative expressions of VEGF and HIF-1α were not affected by 1172 C > T genotypes.

## Discussion

As the largest avascular tissue in the human body, the nucleus pulposus contains cells that are morphologically similar to cartilage cells. The nucleus pulposus is an aggrecan-rich avascular tissue that permits the intervertebral disc to resist compressive loads. The metabolism of such cells is performed by capillary diffusion from the end plate of the cartilage found in their vicinity [24]. HIF-1α is widely expressed in almost all cells in the human body; HIF-1α is also a molecule that detects oxygen stress and cell responses. HIF-1α is degraded under normal oxygen stress and undergoes ubiquitination. However, degradation is suppressed when the cells are exposed to hypoxic condition; as a result, HIF-1α is amplified rapidly and accumulates in cells [25].

An *in vitro* study showed that the oxygen-independent stabilization of HIF-1α in nucleus pulposus cells is a metabolic adaptation that initiates glycolysis and aggrecan expression [16]. Another study has shown HIF-1α expression in the intervertebral disc cells, in which HIF-1α also regulates the apoptosis of intervertebral disc cells, suggesting that HIF-1α is involved in the homeostasis of intervertebral disc cells [26].

The HIF-1α gene is located at chromosome 14q21–q24. *In vitro* studies have demonstrated that the C1772T (P582S) and G1790A (A588T) polymorphisms of the HIF-1α gene under normoxic and hypoxic conditions show a higher transcriptional activity correlated with the overexpression of the corresponding protein than the transcriptional activity and protein expression of the wild-type sequence [27–29]. In clinical practice, the presence of these polymorphisms is associated with the development of diseases such as cancer, diabetes, and coronary disease, in which an altered HIF-1α is observed in various groups among different populations [30–32].

Currently, studies on the association between HIF-1α and LDD have not been published yet. A large-scale replication study of the association of rs17039192 in HIF-2α with knee osteoarthritis (OA) has revealed that rs17039192 in HIF-2α is not positively associated with knee OA [33]. In the present study, the association between SNPs of the HIF-1α gene and the HIF-1α expression as well as the incidence and severity of LDD was analyzed in a Chinese cohort. Significant differences were observed in the allelic and genotypic distributions of the 1790 A > G polymorphisms between the LDD cases and the control subjects. Logistic regression revealed that the 1790 AA genotype represented a protective effect against the development of LDD. To the best of our knowledge, this study is the first to report the function of HIF-1α gene SNPs in LDD. The HIF-1α 1790 A > G polymorphisms also affected the severity of LDD evaluated based on mJOA scores. The 1790 AA genotype carriers exhibited significantly lower mJOA scores than AG and GG carriers. Our study suggested that the HIF-1α 1790 A > G polymorphisms may be used as a molecular marker of the susceptibility and severity of LDD.

VEGF has an important function in physiological and pathological angiogenesis. The upregulation of VEGF expression in response to reduced oxygen stress occurs via transcriptional and post-transcriptional mechanisms [34]. A study in glioma cell has demonstrated that the binding site of HIF1 is important to induce VEGF gene expression under hypoxic conditions [35]. In visceral adipose tissues, hypoxia induces VEGF synthesis [34]. In this study, the HIF-1α gene polymorphisms in herniated lumbar intervertebral disc samples from patients with LDD were investigated. We found that 1790 A > G SNPs, not 1772C > T SNPs, significantly affected HIF-1α and VEGF expressions. VEGF and HIF-1α exhibited similar expression patterns. This result supported our hypothesis that the genetic polymorphisms of HIF-1α in LDD may occur by changing the HIF-1α and VEGF expressions.

A previous study also revealed that HIF-1α expression is strongly correlated with inflammation and angiogenesis in rheumatoid arthritis [36]. We postulated that HIF-1α may regulate the inflammation of intervertebral discs and influence the development of LDD. Our ongoing animal study focuses on the association between HIF-1α expression and inflammation level in intervertebral disc tissues.

## Supporting Information

Table S1 **Characteristics of patients donating samples for western blot assay.**
(DOC)

Table S2 **Genotypes of patients donating samples for western blot assay.**
(DOC)
