# Supplementary material for: Polymorphism in the Hypoxia-Inducible Factor 1alpha Gene May Confer Susceptibility to LDD in Chinese Cohort
Source: PLoS One. 2013 Aug 26;8(8):e73158. doi: 10.1371/journal.pone.0073158 (PMC3753262; doi:10.1371/journal.pone.0073158)
Supplement: Table S1 — Characteristics of patients donating samples for western blot assay. (DOC) [file pone.0073158.s001.doc]

Table S1. Characteristics of patients donating samples for western blot assay.

| Variables Case for western blot |
| --- |
| Age(mean ± SD) 47.4 ± 6.4  Gender (Male, %) 66 (49.3%)  BMI(mean ± SD) 23.2 ± 4.1  Smoking (%) 50 (37.3%)  Family history (%) 19 (14.2%)  History of labor work (%) 47 (35.1%)  DM 28 (20.9%) |
