# Supplementary material for: Polymorphism in the Hypoxia-Inducible Factor 1alpha Gene May Confer Susceptibility to LDD in Chinese Cohort
Source: PLoS One. 2013 Aug 26;8(8):e73158. doi: 10.1371/journal.pone.0073158 (PMC3753262; doi:10.1371/journal.pone.0073158)
Supplement: Table S2 — Genotypes of patients donating samples for western blot assay. (DOC) [file pone.0073158.s002.doc]

Table S2. Gneotypes of patients donating samples for western blot assay.

|  | LDD |  |
| --- | --- | --- |
|  | N=134 | % |
| 1772CC | 45 | 33.58% |
| 1772CT | 72 | 53.73% |
| 1772TT | 17 | 12.69% |
| C | 162 | 60.45% |
| T | 106 | 39.55% |
| 1790GG | 44 | 32.84% |
| 1790GA | 56 | 41.79% |
| 1790AA | 34 | 25.37% |
| G | 144 | 53.73% |
| A | 124 | 46.27% |
